# Transgender people’s knowledge about the adverse effects of cross-hormonization: challenges for nursing

**DOI:** 10.1590/0034-7167-2023-0346

**Published:** 2024-09-20

**Authors:** Andréa Felizardo Ahmad, Adriana Lemos, Cláudia Regina Ribeiro, Luciane Marques de Araujo, Vanessa de Almeida Ferreira Corrêa, Janaína Pinto Janini, Rosâne Mello, Beatriz Rodrigues Silva Selles Dantas

**Affiliations:** I Universidade Federal do Estado do Rio de Janeiro. Rio de Janeiro, Rio de Janeiro, Brazil; II Universidade Federal Fluminense. Niterói, Rio de Janeiro, Brazil; III Universidade do Estado do Rio de Janeiro. Rio de Janeiro, Rio de Janeiro, Brazil; IV Centro Universitário IBMR. Rio de Janeiro, Rio de Janeiro, Brazil; V Secretaria Municipal de Maricá. Maricá, Rio de Janeiro, Brazil

**Keywords:** Knowledge, Adverse Effects, Nursing, Gonadal Steroid Hormones, Transsexualism, Conocimiento, Efectos Adversos, Enfermería, Hormonas Esteroides Gonadales, Transexualidad

## Abstract

**Objectives:**

to identify trans women’s and men’s knowledge about the adverse effects of cross-hormonization and understand the repercussions of hormonization practices on trans women’s and men’s health.

**Methods:**

exploratory, descriptive, qualitative research, developed with 41 participants, from July 2019 to February 2020, in a trans health outpatient clinic. Thematic-categorical content analysis was used.

**Results:**

from the analysis, the categories emerged: Knowledge about the adverse effects of cross-hormonization; and Cross-hormonization practices and their meaning.

**Final Considerations:**

nursing practices, based on the identification of knowledge about adverse effects and the understanding of cross-hormonization practices in trans women’s and men’s health, can result in more inclusive care.

## RESUMEN

**Objetivos:**

identificar los conocimientos de mujeres y hombres trans sobre los efectos adversos de la hormonización cruzada y comprender las repercusiones de las prácticas de hormonalización en la salud de mujeres y hombres trans.

**Métodos:**

investigación exploratoria, descriptiva, cualitativa, desarrollada con 41 participantes, de julio de 2019 a febrero de 2020, en un ambulatorio de salud trans. Se utilizó análisis de contenido temático-categórico.

**Resultados:**

del análisis surgieron las categorías: Conocimiento sobre los efectos adversos de la hormonización cruzada; y Prácticas de hormonización cruzada y su significado.

**Consideraciones Finales:**

las prácticas de enfermería, basadas en la identificación de conocimientos sobre efectos adversos y la comprensión de prácticas de hormonización cruzada en la salud de mujeres y hombres trans, pueden resultar en cuidados más inclusivos.

## INTRODUCTION

The perception of the need to investigate trans women’s and men’s knowledge about the adverse effects of cross-hormonization emerged from the observation, empirically, that part of the transgender population, due to the difficulty in accessing healthcare and seeking to affirm their choices, resorted to dangerous therapeutic resources, one of them being self-medication. Given these facts, it was necessary to research whether transgender people, who use hormones, are aware of the repercussions that these substances can have on their health.

In this context, the transition between genders is what characterizes a transgender person as a person who has a gender identity that is different from the gender assigned according to their genitalia at birth. The agreement between genderba identity and the sex defined at birth designates a cisgender person, also called a cis woman or man^(1)^.

It is noteworthy that the transvestite is also a trans person, as she moves between genders; they were born biologically male and have, in the same body area, feminine and masculine nuances, however transvestites refer to themselves as female and build their body according to that gender^(2)^.

During the 1980s, the HIV/AIDS (Human Immunodeficiency Virus/Acquired Immunodeficiency Syndrome) epidemic strengthened social movements in search of public policies for the LGBT population, and the consolidation of these policies occurred due to the union between members. However, there was reluctance to bring together the homosexual movement and transvestites in the same group, including the letter T in the acronym, as, until then, the movement was called the Brazilian Homosexual Movement, covering only gays and lesbians. In 1995, there was the official participation of transvestites, with the addition of the letter T. The letter, then, referred only to them, as the inclusion of transgender people only occurred in the 2000s^(3)^.

The social representation of transvestites involves the violation of political agreements within the social movement with the inclusion of transgender women, given the social discrepancy between the two. This violation of political agreements is linked, according to transvestites, to the fact that transgender women are less committed to political activism and more concerned with changing their bodies through the transsexualization process^(4)^.

Considering the transition between genders, the hormone indicated for transgender women is estrogen, in order to develop secondary feminine characteristics, the most desired being thin face, obtaining rounded body shapes and breast enlargement, among other body changes. In trans men, testosterone is used, the purpose of which is to form secondary masculine characteristics, the most desired being deep voice, appearance of hair, among others. Hormonization must have careful professional monitoring, given the associated risk factors. Therefore, cross-hormonization is not recommended and adopted only as a step prior to sexual reassignment surgeries, but sometimes it is a process that ends the search for the desired changes^(5-6)^.

The present work is justified by the possibility of qualifying nursing actions based on the identification of transgender people’s knowledge about the adverse effects related to cross-hormonization. Therefore, the relevance of this study for the trans population can be seen through nursing contribution in scientific research and qualified and safe healthcare for transgender people.

## OBJECTIVES

To identify trans women’s and men’s knowledge about the adverse effects of cross-hormonization and understand the repercussions of hormonization practices on trans women’s and men’s health.

## METHODS

### Ethical aspects

This article is an excerpt from the Master’s Dissertation entitled “*Ah! Sei lá, só quero ser eu: significados, saberes e práticas da hormonização cruzada na saúde de mulheres e homens trans*”, presented to the *Escola de Enfermagem Alfredo Pinto* Graduate Program, *Universidade Federal do Estado do Rio de Janeiro* (UNIRIO), Rio de Janeiro, Brazil^(7)^.

In compliance with ethical issues in research with human beings, this study was approved by the Research Ethics Committee, fulfilling all criteria and precautions for carrying out research involving human beings in Brazil, regulated by Resolutions 466/2012 and 510/2016 of the Brazilian National Health Council.

Participants, after being informed about the study justification, objectives and methodology, signed an Informed Consent Form, in clear and objective language, with essential information to facilitate understanding for everyone. Data confidentiality and respect for anonymity were ensured through code names with the letters “W” (transgender woman) and “M” (transgender man), followed by numbers (W1, M2, etc.), according to the order in which the interviews were carried out.

### Study design

This is exploratory, descriptive research, with a qualitative approach. The study was conducted in accordance with the COnsolidated criteria for REporting Qualitative research (COREQ) reporting guideline^(8)^.

### Study setting

The study was carried out in a trans health outpatient clinic, part of a public service polyclinic in the city of Niterói, RJ, Brazil. The geographic factor was the reason for choosing this setting. In the aforementioned outpatient clinic, a social worker, an endocrinologist and a psychologist provide assistance to the transgender population. It must be emphasized that the authors of this study do not have any connection with the institution in the study setting, however the main researcher has experience in welcoming trans people.

### Data source

A total of 13 transgender women and 28 trans men participated in this research, and none of the participants identified themselves as transvestites. Trans women and men over the age of eighteen and who were users of the aforementioned outpatient clinic, whose selection occurred in a simple random, were included. People who identified as non-binary were excluded from this study, as they did not meet the objectives of this investigation.

### Data collection and organization

Firstly, an approach to the setting was carried out, through a technical visit, in which dialogues took place with professionals working in the study setting and with study users. Fieldwork began in July 2019, and ended in February 2020.

To obtain the data, participants were taken to a private room, where semi-structured interview took place, using a data collection instrument with 11 questions pertinent to the objectives of this study, in addition to questions relating to participant characterization. The interviews were audio recorded and later transcribed.

### Data analysis

To process the data, thematic-categorical content analysis was used, starting with a skimming reading of all interviews and then provisional hypotheses were constructed^(9-10)^. After transcribing each interview, these were completed in number 41, at which time the absence of new phenomena was noted and data robustness was perceived, in order to achieve the proposed objectives.

After skimming the interviews, Registration Units (RU) were created according to interviewees’ statements, totaling 141 RU, and, after thematic analysis, they were grouped into 19 Meaning Units (MU), according to the themes found.

## RESULTS

Participants’ median age was 24 years old. In Chart 1, it is possible to observe participant socioeconomic and demographic characteristics.

**Chart 1:** Research participant socioeconomic and demographic characterization

Characteristics	N = 41	Percentage
**Gender**		
Transgender woman	13	32%
Transgender man	28	68%
**Sexual orientation**		
Heterosexual	21	51%
Pansexual	08	20%
No sexual orientation	04	10%
Bisexual	03	07%
Homosexual	01	02%
Did not want to answer	04	10%
**Education**		
Complete high school	17	41%
Incomplete higher education	13	32%
Incomplete high school	04	10%
Complete elementary school	02	05%
Incomplete elementary school	02	05%
Complete higher education	01	02%
Did not want to answer	02	05%
**Self-reported race/ethnicity**		
Black	27	66%
White	11	27%
Did not want to answer	03	07%
**Religion**		
No religion	23	56%
Catholic	06	15%
Umbanda	05	12%
Candomblé	04	10%
Evangelical	01	02%
Did not want to answer	02	05%
**Marital status**		
Single	27	66%
Stable union	07	17%
Married	06	15%
Separated	01	02%
**Family income**		
< than a minimum wage	01	02%
01 to 02 minimum wages	07	17%
02 to 04 minimum wages	07	17%
> 04 minimum wages	02	05%
Variable income	05	12%
Did not want to answer	19	46%

It was observed that, regarding sexual orientation, heterosexuality predominated. With regard to education, the majority of participants reported that they completed high school. Regarding race/ethnicity, black race self-declaration prevailed. Regarding religion, the largest number of participants said they had no religion. Regarding marital status, the majority called themselves single, and regarding family income, there was a predominance of income of up to four minimum wages.

There was a diversity of occupations/professions among participants; however, it should be noted that 29.82% reported not having any occupation at the time of the interview.

It should be noted that tobacco consumption was mentioned by 50% of trans men, and 15.38% of transgender women reported using the substance.

From the articulation of participants’ speeches, the following categories were created:

### Category 1 - Knowledge about the risk factors of cross-hormonization

This category was constructed from the articulation of 84 RU from the analysis *corpus*, originating 16 MU, with emphasis on the risk of cardiovascular problems and the risk of developing cancer. Regarding the adverse effects of cross-hormonization, 78% of participants declared that they knew some effects, but some people did not show concern about these possible damages:

*We, in the trans world, have a lot of this, of recommending ourselves to each other, you know. The last thing that comes to mind is that it could be harmful.* (W4)

*I started when I was 16, taking, I don’t know, one ampoule a week, two, I don’t know* [...] *I just wanted a quick result, and what I always read from the beginning when I started studying is that we have to remove the uterus or ovary, because this can cause cervical cancer, that’s what they say.* (M20)

*How do I go to a doctor? He won’t recognize me as a trans man* [...] *I’ve already had such bad cramps, I cried in pain* [...]*, and the doctor says, a big guy like that, crying in pain* [...] *so much pain, that my blood pressure even increased, and I’m hypertensive!* (M24)

Regarding the risk of developing cancer, its correlation with cross-hormonization was highlighted as a concern, as seen in the statements below:

*I was afraid of starting the transition and having breast cancer, uterine cancer and so on.* (M09)

*Some friends of mine had this cancer thing* [...] *then I got scared, I found out that it could cause cancer* [...] *in our* [penises]. (W01)

It should be noted that, even though the trans body has some singularities, gender identity cannot be considered an impediment to healthcare so that reception and care for all people must occur in an equal and equitable manner. Despite this, there are trans men who mention different reasons for not seeking gynecological care, as noted below:

*I was always terrified of going to the gynecologist. I went, but, in that first contact, I felt a little lack of empathy, lack of knowledge* [...] *in some cases, even a little discrimination* [...] *so I simply got up from my chair and left, I didn’t I wanted to be assisted.* (M09)

*I don’t want to, because I can barely look at myself in the mirror, why, right? I am different. For other people to see what I don’t want to see, for me, it would be even more of a problem. And nowadays, I don’t know if I’ll ever be able to see a gynecologist in my life.* (M04)

Given the facts reported, it is understood that, through the link between transgender people and the health service, it is possible to identify early the adverse effects of cross-hormonization that can result in damage to health.

### Category 2 - Cross-hormonization practices and their meaning: “I want to be able to look at myself and see myself the way I imagine myself!”

This category represented a total of 57 RU in the analysis *corpus*, grouped into three MU: no previous hormone treatment; hormone therapy without a medical prescription; and hormone therapy with medical prescription. All people interviewed declared the desire for cross-hormonization and of the total number of participants, and 19 reported never having used hormones on their own, 4 being transgender women and 15 trans men. Of those who used it, 13 reported using it without a medical prescription and 14 started or planned to start hormone therapy with a prescription.

Some trans men were emotional after receiving the medical prescription to start cross-hormonization, as can be seen in the following statements:

*Oh, I have no words! I don’t think I ever thought that pieces of paper would make me so happy.* (M01)

*Wow, it was a lot of happiness, because I had been waiting for this for a long time, you know, in that anxiety of starting my therapy, the transformation, you know?* (M05)

For transgender women, access to hormone therapy had a different meaning, as they were happy to receive the prescription, but without the explicit euphoria and joy of men, seen in the statements below:

*Now I’m going to start taking the medication the doctor prescribed.* (W02)

*I came to see the endocrine specialist to do hormonal therapy and now I’m looking for a psychologist.* (W08)

This fact may be related to easier access to hormones, as the acquisition of female hormones involves first searching for information, especially through other transgender women:

*There, no one prescribes or gives medication to anyone. It’s an exchange of knowledge, oh, I took the medication. Others undergo endocrine treatment, “Ah, my endocrine system gave me that”. It’s an exchange of experience, in fact. But then, crazy as I am, I went and thought, “So-and-so took this and it was cool, I’ll take that too”.* (W09)

In the case of men, even with the difficulties caused by the fact that testosterone can only be acquired with a medical prescription, there are transgender men who are able to acquire this hormone over the internet or in gyms, as we see in the speeches below:

*I went* [online], *and I started to see that trans men who had the same body type as mine and how long they took the medication, and I started doing the same cycle.* (M09)

*I did hormone treatment at home. I bought it at a gym, because there was no clinic, I didn’t have access to that.* (M22)

Regarding the use of hormones without a medical prescription, six transgender women and 15 trans men mentioned that they had never used hormones on their own, even though they had easy access:

*I never liked these things* [self-medication]*, because I was always mature in that sense. I never liked taking medication without a doctor’s recommendation.* (W06)

The risks of using hormones without medical supervision are highlighted, as the harm to health may outweigh the benefits expected by the transgender population.

## DISCUSSION

Resolution 47 of the Collegiate Board (RDC) of the Brazilian National Health Regulatory Agency (ANVISA - *Agência Nacional de Vigilância Sanitária*) of September 8, 2009 defines an adverse effect as “any unfavorable medical occurrence, which may occur during treatment with a medication, but which does not necessarily has a causal relationship with this treatment”^(11)^.

In this context, the Ministry of Health (MoH) is emphatic about educating the trans population regarding hormone treatment, its prolonged use and possible adverse effects. In this way, the MoH aims to reduce risks and damages caused by the indiscriminate use of medications (including hormones) among the trans population. Continuous information and periodic monitoring must be offered in order to minimize risks and protect the transgender population from harm to their health^(12)^.

Regarding the risk of developing cardiovascular problems, thrombosis was mentioned by some participants, showing concern about this imminent danger. It should be noted that one of the risk factors for cardiovascular diseases, including thrombosis, is tobacco consumption, which was cited by a portion of the research participants. Smoking is one of the main causes of cardiovascular diseases and the most varied types of cancer. The ease of obtaining the drug, due to its legality and low cost, allows its consumption to be unrestrained, even if information on comorbidities is provided^(13)^.

However, the risk of cardiovascular diseases is not only related to risk factors, such as alcohol and tobacco consumption, but also to cross-hormonization related to these factors, as these substances, associated with the use of testosterone and estradiol valerate, may increase the risk of venous thromboembolism and heart disease. Stress is also a risk factor for cardiovascular disease^(14)^.

Therefore, it is understood that regular monitoring by health professionals, given the possible adverse effects that may occur during cross-hormonization, can contribute to reducing cardiovascular risk factors^(15)^.

Regarding the risk of developing cancer, an Australian study showed the ineffectiveness of cervical cancer screening in trans men due to several factors, from professional inability to carry out the test to trans men’s embarrassment when seeking the procedure, due to discrimination and dysphoria in relation to genitalia^(16)^.

It should be noted that trans men should not be excluded from collecting the cytopathological test (the collection of this test being generally optional in trans men who have never had sexual intercourse with penile penetration), highlighting that this test aims to detect the presence of precursor lesions of cervical cancer as early as possible^(2)^.

Regarding the incidence of breast cancer in trans men, its occurrence is rare, but they must undergo screening for the disease, as recommended, including trans men who have had a mastectomy, just as the screening test for breast cancer in transgender women^(17)^.

The discussion about the importance of screening for cancer prevention is highlighted by the incidence of various cancers, such as breast neoplasms, neoplasms related to the use of androgens by trans men (even those who have had a mastectomy) and prostate cancer, associated with estrogen use by transgender women. Thus, more studies are needed to confirm the hypotheses formulated^(18)^.

It is understood that prejudice and pre-judgments keep nurses and other health professionals away from providing care in a welcoming and resolute manner, thus compromising trans people’s health with regard to health education related to the adverse effects of cross-hormonization. This behavior is a form of violence that contributes to some diseases not being properly tracked, due to the vulnerability to which the trans population is exposed^(19)^.

These aspects corroborate how relevant health education is, given the inconsistency in obtaining information and discrimination, already implicit in the transgender population. Therefore, it is essential to train health professionals regarding the trans population’s health and, in this way, provide a welcoming and respectful environment for providing care to this population^(20)^.

In terms of health promotion, the barriers to be overcome include the lack of interest of managers in training health professionals and some nurses’ inertia in proposing health actions based on problems that users could bring, including doubts about cross-hormonization^(21)^.

In order for these demands to be perceived, the bond between users and nurses is essential, as is the understanding of the LGBT Healthcare Policy, which addresses several issues, including cross-hormonization^(11)^.

In this context, health education, not only for the transgender population, but also for society as a whole, can be a game changer in reducing discrimination and prejudice, factors that make it difficult for trans women and men to access health services.

A study carried out in 2017, in Brazil, observed that, of the ten transgender women participating in the research, eight used cross-hormonization without a medical prescription, thus realizing the existence of changes in behavior and implications for fertility^(22)^.

Regarding the use of hormones without a medical prescription, the reasons given by the participants in the present study for this practice were the difficulty in accessing health services and the desire for quick results. Thus, it is necessary to reflect on the fact that more transgender women than trans men use cross-hormonization without a medical prescription, facilitated by the possibility of purchasing female hormones without a proper prescription.

Furthermore, obtaining female hormones often occurs after considerations obtained on the internet and through other transgender women. However, the bodily changes caused by cross-hormonization are not enough given the social barriers that transgender women experience^(23)^.

Barriers to accessing health services and, consequently, to hormonal prescription, encourage the search for inappropriate information and the initiation of cross-hormonization indiscriminately, putting their health at risk^(24)^.

In light of the issues presented, it is understood that hormonization is the vehicle for quickly obtaining, for some, the feminine or masculine forms, which are so important for trans women’s and men’s individuality, which often leads to use without medical supervision, even if the adverse effects are known. However, for participants in this research, this stance was not the predominant one.

### Study limitations

As a limitation of this study, it is worth highlighting the fact that the health unit, which was the study setting, only operates once a week, which entailed logistical issues regarding the time for data construction to occur. However, the results presented corroborate the importance of discussing the topic in healthcare spaces, reflecting the need for more studies that address issues related to transsexuality.

As another limitation of this study, the absence of nursing professionals in the study setting stands out, given the healthcare contributions, highlighting that nursing is part of the multidisciplinary team of the transsexualization process. Furthermore, nursing care practices, based on transgender people’s knowledge about cross-hormonization, can be implemented in comprehensive and equitable care and in health educational actions regarding the importance of cross-hormonization with monitoring by a multidisciplinary team.

### Contributions to nursing, health or public policy

Through this study, it was possible to reflect on the peculiarities that trans women and men face, in order to obtain healthcare and have their rights respected. The results of this study demonstrated that trans populations’ knowledge about cross-hormonization and cross-hormonization practices on trans population’s health are factors that significantly impact nursing care and healthcare for this public, through the construction of therapeutic projects and creation of bonds, and in terms of prevention and early detection of adverse effects caused by hormone therapy, in addition to cancer screening.

## FINAL CONSIDERATIONS

This study provided, through participants’ narratives, the identification of trans women’s and men’s knowledge about the adverse effects of cross-hormonization, highlighting that the majority of participants declared that they knew the adverse effects of cross-hormonization through internet research and information shared in trans discussion groups.

This knowledge did not prevent some people from using cross-hormonization without health professionals’ supervision; however, it is noteworthy that the search for healthcare in a comprehensive health clinic for the trans population demonstrates the concern of these people with their health, in order to enjoy the maximum benefit of hormonization, and that the adverse effects and risks inherent to hormonization could be quickly identified, although some have found easier and faster ways to practice cross-hormonization through self-medication.

Unfortunately, it is not possible to reliably predict the adverse effects that cross-hormonization may cause in each person, as factors such as smoking and the use of these substances without professional supervision may obscure some risks, such as the development of cancer and cardiovascular problems. Therefore, careful monitoring by the multidisciplinary team is necessary, in order to prevent these possible effects and provide full health and satisfaction for each person in their gender identity.

This study allowed us to understand the repercussions of cross-hormonization practices on trans women’s and men’s health, as the search for a body corresponding to the interests of trans women or men through cross-hormonization is a demand characterized by several common meanings: travel through the most diverse spaces (including health) without facing discriminatory looks or prejudiced comments; look at oneself and like what they see, recognizing oneself and being recognized or recognized according to gender identity, in the face of the acquisition of secondary characters; and still obtain peace of mind in their daily life. Another important repercussion was the security offered by passing in the face of transphobia often faced by transgender people.

Finally, the contribution of this study to promoting research in the field of healthcare for the transgender population stands out as well as to undergraduate education not only in the area of nursing but also in health in general. The discussion about transsexuality in healthcare spaces, both through extension activities, in partnership with universities, and through continuing education, can contribute to more inclusive professional practices and thus, contribute to the reduction of transphobia that causes so much harm to this population’s health. In this way, this study contributes to minimizing risks and damages given the social vulnerability to which this public is exposed.

## Supplementary Material

0034-7167-reben-77-04-e20230346-suppl01

0034-7167-reben-77-04-e20230346-suppl02

0034-7167-reben-77-04-e20230346-suppl03

0034-7167-reben-77-04-e20230346-suppl04

0034-7167-reben-77-04-e20230346-suppl05

0034-7167-reben-77-04-e20230346-suppl06

## Data Availability

*https://doi.org/10.48331/scielodata.HWNTYR*
