# Supplementary material for: Transgender people’s knowledge about the adverse effects of cross-hormonization: challenges for nursing
Source: Rev Bras Enferm. 2024 Sep 20;77(4):e20230346. doi: 10.1590/0034-7167-2023-0346 (PMC11419685; doi:10.1590/0034-7167-2023-0346)
Supplement: 0034-7167-reben-77-04-e20230346-suppl01 [file 0034-7167-reben-77-04-e20230346-suppl01.pdf]

APÊNDICE E

CARACTERIZAÇÃO DOS PARTICIPANTES

Part. [1]	Idade	IG [2]	OS [3]	Raça/ Etnia	Escolaridade	Profissão	Estado Conjugal	Renda (em SM) [4]	Religião
H01	35	Homem Trans	Heterossexual	? [5]	?	Comerciante	Casado	?	?
H 02	?	Homem Trans	Pansexual	Branco	Médio Incompleto	Jovem Aprendiz	União estável	02 a 03	Umband
M 01	20	Mulher Trans	Heterossexual	Parda	Fundamental Incompleto	Cabeleireira	Solteira	1	Não ten
NB 01	34	Não binário	Assexual	Branca	Superior Incompleto	Trabalha em Biblioteca	Solteira	3	Não ten
H 03	18	Homem Trans	Heterossexual	?	Médio Incompleto	Desempregado	União estável	2	Não ten
H 04	22	Homem Trans	Não definido	Branco	Médio	Free lancer	Solteiro	Variável	Não ten
H 05	30	Homem Trans	Heterossexual	Pardo	Fundamental Incompleto	Auxiliar de Produção	União estável	1	Católico
H 06	19	Homem Trans	Não definido	Branco	Superior Incompleto	Estudante	Solteiro	Variável	Não ten
M 02	31	Mulher Trans	Homossexual	Parda	Médio	Cabeleireira	Solteira	1,5	Não ten
H 07	32	Homem Trans	Heterossexual	Pardo	Médio	Desempregado	Solteiro	1	Não ten
H 08	28	Homem Trans	Heterossexual	Negro	Médio	Técnico em Enfermagem	União estável	2	Umband
M 03	24	Mulher Trans	Heterossexual	Parda	Médio	Desempregada	Casada	1	Não ten
M 04	22	Mulher Trans	Pansexual	Branca	Médio	Vendedor de desenhos	Solteira	1	Não ten
H 09	28	Homem Trans	Heterossexual	Branco	Superior Incompleto	Desempregado	Solteiro	?	Umband
H 10	23	Homem Trans	Heterossexual	Negro	Superior Incompleto	Free lancer	Solteiro	Variável	Evangéli
H 11	21	Homem Trans	Não definido	Branco	Médio	Desempregado	Solteiro	Variável	Não ten
M 05	27	Mulher Trans	?	Branca	Fundamental	Cabeleireira	Solteira	< 01	Não ten
M 06	22	Mulher Trans	?	Negra	Médio	Desempregada	Solteira	?	Não ten
H 12	?	Homem Trans	?	?	?	Vendedor	Casado	?	?

H 13	23	Homem Trans	Pansexual	Pardo	Médio	Trabalha com alimentos	Solteiro	2	Não ten
H 14	22	Homem Trans	Pansexual	Negro	Médio	Estudante	Solteiro	02 a 03	Não ten
H 15	28	Homem Trans	Heterossexual	Branco	Superior Incompleto	Desempregado	União estável	02 a 03	Não ten
H 16	25	Homem Trans	Heterossexual	Negro	Superior Incompleto	Estudante	Solteiro	2	Católico
H17	19	Homem Trans	Heterossexual	Negro	Superior Incompleto	Pensionista	União estável	1	Não ten
H 18	26	Homem Trans	Heterossexual	Negro	Médio	Balconista	Casado	3	Umband
M 07	32	Mulher Trans	Heterossexual	Parda	Médio	Desempregada	Solteira	Variável	Não ten
H 19	19	Homem Trans	Bissexual	Branco	Superior Incompleto	Professor voluntário	Solteiro	Variável	Não ten
M 08	32	Mulher Trans	Não definido	Negra	Superior Incompleto	Desempregada	Solteira	5	Não ten
H 20	24	Homem Trans	Pansexual	Negro	Médio	Ator	Solteiro	Variável	Umband
H 21	23	Homem Trans	Bissexual	?	Superior Incompleto	Estagiário	Solteiro	?	Candomb
M 09	25	Mulher Trans	Heterossexual	Negra	Médio	Atendente	Casada	3	Católica
H 22	20	Homem Trans	Heterossexual	Negro	Médio	Barbeiro	Casado	2	Não ten
H 23	20	Homem Trans	Pansexual	Branco	Superior Incompleto	Professor voluntário	Solteiro	1	Candomb
H 24	31	Homem Trans	Pansexual	Negro	Superior Incompleto	?	Solteiro	1,4	Não ten
M 10	25	Mulher Trans	Heterossexual	Branca	Médio	Garçonete	Solteira	1,5	Não ten
H 25	19	Homem Trans	Bissexual	Negro	Médio Incompleto	Desempregado	Solteiro	1	Candomb
H 26	25	Homem Trans	Heterossexual	Negro	Superior Incompleto	?	Solteiro	2,5	Agnóstic
M 11	20	Mulher Trans	Heterossexual	Negra	Médio	Estudante	Solteira	1	Católica
M 12	22	Mulher Trans	Heterossexual	Parda	Fundamental	Cabeleireira	Solteira	1,3	Católica
M 13	56	Mulher Trans	?	Parda	Superior	Desempregada	Separada	Sem renda	Não ten
NB 02	24	Não binário	Assexual	Amarela	Médio	Desempregado	Solteiro	1	Não ten
H 27	18	Homem Trans	Pansexual	Negro	Médio Incompleto	Vendedor	Solteiro	< 01	Candomb
H 28	41	Homem Trans	Heterossexual	Negro	Superior Incompleto	Assistente administrativo	União estável	5	Não ten

Legenda:

1 - Participante

2 - Identidade de Gênero

3 - Orientação Sexual

4 - Salário Mínimo

5 - o participante não soube ou não informou
