# Supplementary material for: Transgender people’s knowledge about the adverse effects of cross-hormonization: challenges for nursing
Source: Rev Bras Enferm. 2024 Sep 20;77(4):e20230346. doi: 10.1590/0034-7167-2023-0346 (PMC11419685; doi:10.1590/0034-7167-2023-0346)
Supplement: 0034-7167-reben-77-04-e20230346-suppl02 [file 0034-7167-reben-77-04-e20230346-suppl02.pdf]

Fala / Unidades de Registro (URs)	Part.	US	Total
“- Ele é francês, o cara que me vende, minha namorada é fluente em francês, então eu confiei no que ela leu.” “- Tomei três meses e não vi resultado”.	H2	Hormonização sem prescrição medica	2
“- ...comecei a tomar os meus remédios com quinze anos de idade e foi indo...” “- Eu fui comprando na farmácia.... Por conta própria... Com uma amiga minha que é travesti.” “- Usei o Perlutan... Só teve efeito positivo.” “-Eu tomei o Perlutan e parei, por falta de dinheiro.”	M1		4
“– E você já tomou alguma medicação sem prescrição? – Já, tomei Perlutan.”	M2		1
“– Você já usou hormônio antes? – Já, comecei com Durateston, mas foi pouquinho...sozinho.”	H7		1
“ – Já tomou sem receita? – Já.”	M3		1
“- ...comecei sem a prescrição...A maioria dos transexuais começam o tratamento por conta própria.”	H9		1
“- Tomou por conta própria por quanto tempo?- Por uns três meses.”	M5		1
“- Você já usou hormônio alguma vez? - Já, várias vezes!” “- Então, eu comecei...tomando sei lá, uma ampola por semana ,duas sei lá…só queria um resultado rápido.”	H20		2

"- ...eu fiz tratamento durante 3 anos da minha vida, tratamento hormonal, por conta própria, né?"	M9		1
"-...eu fazia tratamento hormonal em casa mesmo, eu comprava em academia..."<br><br>"- ...por que eu tava fazendo o tratamento hormonal em casa".	H22		2
"-...a hormonização que eu faço é por conta própria. "<br>"- E onde que você buscou informações para saber que hormônio utilizar? - Internet… "	H24		2
"- Comecei a tomar por conta própria!"	M12		1
"-Foi por conta própria? - Foi sim…"	M13		1
		TOTAL DE URS	2[illegible]
		Nenhuma hormonização anterior	
"- Nunca tinha tomado hormônio?- Não. Nunca."	H4		1
" - E alguma vez na vida você usou hormônio por conta própria? - Não. Quase."	H6		1
"- Já fez algum tratamento de hormônio antes? - Não."	H8		1
"- Não, eu sempre tive medo de tomar por conta própria."	M4		1
"– Nunca usou homônio? – Não."<br><br>" – Você não começou a usar testosterona ainda? – Não."	H10		2

"– Já usou alguma vez, por conta própria?– Não."	H11		1
"- Antes de você vir aqui, utilizava hormônios por conta própria? - Eu nunca gostei de me medicar sem recomendação médica."	M6		1
"- Já usou hormônio? - Não."	H13		1
"- Já tomou hormônio antes?- Não."	H14		1
"- Já tinha usado hormônio?- Não."	H16		1
"-Porque eu não quero fazer por conta própria."	H18		1
"-Não, não, não fiz. "<br>" - Tenho pavor de qualquer tipo de automedicação."	M7		2
"- E você já usou alguma vez hormônio por conta própria? - Não."	H19		1
"- Fez hormônio alguma vez por conta própria? - Não. "	M8		1
"- Eu não queria começar minha T sem o acompanhamento de um endócrino."	H21		1
" - Não, tenho muito medo de tomar por conta própria."	H25		1
"- Você já usou hormônio por conta própria alguma vez?- Não."	H26		1
"- Eu não tava fazendo terapia hormonal."	NB2		1
" - Já utilizou alguma vez hormônio? – Não. "	H27		1
"- Você utilizou alguma vez hormônio, por conta própria? – Nunca. "	H28		1
		TOTAL DE URS	2[illegible]

		Hormonização com prescrição médica	
“- Chegou à consulta... solicitando receita para voltar a tomar Deposteron”	H1		1
“- ...sair daqui e ir direto a uma farmácia!”	H2		1
“- Não... comecei a usar aqui.”	NB1		1
“- Eu vim aqui porque eu queria que eles me dessem aquele, a receita pra eu poder começar a tomar a T.”	H4		1
“-Vou passar pela primeira consulta e pretendo sair daqui hoje com a minha receita”.	H5		1
“-...agora que vou começar a tomar o medicamento que o médico receitou.”	M2		1
“- Pretendo ir no endocrinologista pra fazer hormonização.”	H14		1
“-Você já fez alguma terapia hormonal antes?- Fiz. Fiz com a Dra Karen. “	H15		1
“- Eu vim procurar o endócrino pra fazer a terapia hormonal.”	M8		1
“- Eu vim buscando esse acompanhamento, porque eu não queria começar minha T sem o acompanhamento de um endócrino. “	H21		1
“-...e comecei a me hormonizar. – Por conta própria? – Não, aqui.”	H23		1
“– Antes dela, você já tinha tomado hormônio por conta própria? – Não, não, eu comecei com o endócrino”	M10		1

“- E você veio buscar o que aqui? - Terapia hormonal, acompanhamento de endócrino!”<br><br>“- Mas você usou por conta própria? - Não pelo endócrino, eu paguei particular.”	M11		2
“- E quem prescreveu esse hormônio?- Quem me passou foi...os dois endócrinos. “	M12		1
		TOTAL DE URS	1[illegible]
		Conhecimento / presença de oscilações de humor	
“- O que eu vi colateral foi mudança de humor só.”<br>“- Uma mudança de humor extrema.”	H2		2
“- Assim, eu sei que, né, vai mudar tudo..tudo né...tanto no humor quanto no corpo.”	H3		1
“- ...risco de mexer um pouquinho com o psicológico, alterações de humor.”	H5		1
“...mudança de humor...algumas coisas te irritam...dá um pouco de irritabilidade.”	H7		1
“- foi mais oscilação de humor.”	H21		1
		TOTAL DE URS	6
		Estresse	
“-Eu to muito estressado... esses hormônios estão mexendo muito comigo.”<br><br>“- É um estresse, assim é muito estresse.”	H8		2

“- ...porque eu vivia muito estressado.”	H9		1
“- fiquei mais estressada...”	M10		1
		TOTAL DE URS	4
		Conhecimento sobre câncer	
“-Tem umas amigas minha que teve esse negócio de câncer... aí eu fiquei com medo, fiquei sabendo que pode dar câncer... nos negócios da gente.”	M1		1
“-...pode até dar coisa do tipo câncer.”	H4		1
“-...por exemplo desenvolver um câncer de mama...”	H7		1
“- ...eu fiquei com medo de iniciar a transição e ter lá pra frente algum câncer de mama, de útero e tal.”	H9		1
“-...o médico já tinha explicado que poderia me dar um câncer.”	M5		1
“-...isso pode dar câncer no colo do útero.”<br>“-...é que a gente tem que retirar na verdade o útero ou ovário porque isso pode dar câncer no colo do útero...”	H20		2
		TOTAL DE URS	7

		Problemas Cardiovasculares	
“- Então, eu sei o risco da trombose, né?”<br>“...-eu descobri que tinha um riscozinho: trombose, cistos, então eu fiquei com receio.”<br>“-...como problemas cardíacos que pode desenvolver.”	H5		3
“-...risco de ter uma trombose......risco também de ter um AVC.”	M2		1
“-...o hormônio que eu tava usando... meu sangue, ele engrossou de mais. Eu tenho uns caroços na perna pelo uso de hormônio.”	M3		1
“-...que pode causar uma trombose.”	M7		1
“- Trombose...”	M9		1
“- Ai, alguns tipos de doenças, né? Que pode dar. Trombose é uma.”	H15		1
“- ...pode dar trombose.”	H24		1
		TOTAL DE URS	9
		Cefaleia	
“ - Tive mais dor de cabeça, aumentou a dor de cabeça.”<br>“– É...enjôo, dor de cabeça...”	M2		2

"-...eu tive muita enxaqueca."	H21		1
		TOTAL DE URS	3
		Depressão	
"-...eu sei assim... pode causar depressão..."	NB1		1
"-...tem dia que a minha mente ta muito perdida, eu me sinto numa depressão, sabe..."	H8		1
"- Então, só depressão mesmo."	H24		1
		TOTAL DE URS	3
		Infertilidade	
"- Sei alguns....tipo, infertilidade..."<br><br>"- Eu fiquei um tanto receoso quanto à infertilidade."	H23		2
		TOTAL DE URS	2
		Enjôo	
"– É...enjôo, dor de cabeça..."	M2		1
"- ...muito enjôo..."	H21		1

		TOTAL DE URS	2
		Alterações cutâneas	
“- Que eu me lembre... a pele fica oleosa.”	H4		1
“- Muita acne...”	H7		1
“-...ele fica com muito pus, pus mesmo, sabe, fica aquela formação feia...parece furúnculo.”	H8		1
“- Começou a me dar muita oleosidade na pele. “	H16		1
“- Ah, espinha...”	H17		1
“-...ah, espinha, voltou bastante.”<br>“-...minha pele ficou mais oleosa...”	M10		2
		TOTAL DE URS	7
		Aumento do apetite	
“-Meu apetite aumentou demais, eu comecei a comer muito.”	H6		1
“-...as vezes eu tinha falta de apetite, as vezes tinha muita fome…”	H9		1
“- ...a T(testosterona) me dá muita fome.”	H21		1

		TOTAL DE URS	3
		Aumento de peso	
“-... eu sei assim... que aumenta a gordura nas áreas do corpo também.”	NB1		1
“- Ganhei peso.”	H16		1
		TOTAL DE URS	2
		Calor	
“- Calor. Muito calor.”	H6		1
“-...suor excessivo...”	H7		1
“-...senti um calor que batia assim...nossa...”	M10		1
“- E sentia muito calor...”	M11		1
		TOTAL DE URS	4
		Alopécia	
“-Que eu me lembre... calvície...”	H4		1

“- Meu cabelo caiu.”	H16		1
“-... Perda de cabelo...”	H18		1
“- Só calvície, uma entrada apareceu...”	H23		1
		TOTAL DE URS	4
		Alterações nos pelos	
“- Falava que podia aumentar o pêlo também aí falava que podia cair. “	NB1		1
“-...tem alguns lugares no corpo que começam a crescer pelo que antes não crescia.”	H4		1
“- Tô com muito mais pelo também.”	H6		1
		TOTAL DE URS	3
		Alterações na libido	
“- Mudou um pouquinho só.”	M1		1
“-...eu sei assim que abaixa a libido.”	NB1		1
“- Assim a libido abaixou consideravelmente.”	M2		1

“-...ela aumenta muito o desejo sexual...a testosterona.”	H7		1
“-...eu acho que minha libido, ela tá muito alta.”	M3		1
“- Mudou a libido? - Mudou a beça.”	H21		1
“- Mas aí minha libido foi lá embaixo.”(TH inadequada).	M9		1
“- Libido? – Melhorou, né...”	H23		1
“-...diminuição da libido, foi bem forte em mim.”	M10		1
“- Então você usando hormônio não tinha tesão, parava de usar o hormônio o tesão voltava? - Hamram!”	M12		1
		TOTAL DE URS	1[illegible]
		Reprodução natural	
“-...Adotando ou até mesmo gerando, talvez...”	H11		1
“- A forma, que você “tá” pesando em ter esse filho, é você engravidando?- Sim.”<br>“- Então você quer engravidar, é isso?- Sim. Daqui pra frente...”	H17		2
“-...mas hoje em dia eu já penso em gerar. “	H20		1

"- Não sei se eu vou engravidar não, talvez."<br><br>"- Mas poderia engravidar? É uma possibilidade? - Poderia."	H21		2
"- Não me vejo, mas gestaria."	H23		1
"-Você pensa em engravidar então? - Penso em engravidar."	H24		1
"-...quero ter filho... gerar o filho."	H25		1
		TOTAL DE URS	9
		Reprodução Assistida	
"- A gente quer adotar e ter por inseminação, entendeu."<br><br>"-...eu tenho esse desejo de fazer a inseminação com a minha namorada."	H2		2
"-...por inseminação ou adotar. Tenho muita vontade de ter filho."	H3		1
"- De mim não. Mas gostaria muito que minha namorada pudesse ter. "	H5		1
"-...se eu tivesse a oportunidade, o capital… congelar uns óvulos para ter filhos…"	H9		1
"- Mas adotar também...inseminação também, com alguém que esteja comigo...menos engravidar."	H10		1
"- Inseminação. Ia fazer inseminação."	H15		1

“-...mas se eu não puder, eu adoto ou se eu estiver com alguém que queira gestar...”	H23		1
“ – Não, eu quero que minha mulher tenha, eu não.”	H28		1
		TOTAL DE URS	9
		Adoção	
“- A gente quer adotar e ter por inseminação, entendeu.”	H2		1
“-...por inseminação ou adotar. Tenho muita vontade de ter filho.”	H3		1
“- Eu não quero, de verdade, ter filho. Só se, por exemplo, for adotado, mas saindo de mim eu não quero não.”	H4		1
“-...eu pensei em adoção...adoção.”	H7		1
“- Mas adotar também...inseminação também, com alguém que esteja comigo...menos engravidar.”	H10		1
“-...Adotando ou até mesmo gerando, talvez.”	H11		1
“-...eu sempre tive na cabeça de adotar.”	H12		1
“- Adotando, mas em mim mesmo não.”	H13		1

"-...então acho que agora eu vou adotar."	H14		1
"- Eu tenho vontade de adotar uma criança. "	H18		1
"- Talvez eu tenha um dia se adotar."	M8		1
"-...mas se eu não puder, eu adoto ou se eu estiver com alguém que queira gestar..."	H23		1
"- Hoje em dia eu só teria adotado mesmo. Eu não queria gerar."	H26		1
		TOTAL DE URS	1[illegible]
		Desinteresse por reprodução	
"- Não, eu não quero ter filhos."	M1		1
"- Pretende ter (filhos)? - Não."	H6		1
"- Não, eu não tenho e nunca tive desejo de ter filhos."	H8		1
"- Não, não penso em filhos."	H19		1
"- Você pretende em ter filhos?- Não!"	M11		1
"- Adoro criança, mas quero não!"	M13		1

		TOTAL DE URS	6
		Demanda por prótese mamária	
“- Quero fazer mama e genital.”	M1		1
“- Ah....quero prótese mamária e da redesignação sexual.”	M2		1
“-...eu pretendo fazer a mudança de sexo...pretendo botar prótese nos seios.”	M3		1
“-...eu tenho vontade mesmo de fazer é a minha prótese (mama) e dar uma modeladinha no meu corpo.”	M9		1
“- Pretendo mexer no meu rosto, no meu queixo principalmente, eu acho muito masculino...e colocar prótese.”	M10		1
“- Quero fazer silicone.”	M11		1
		TOTAL DE URS	6
		Demanda por redesignação sexual	
“- Quero fazer mama e genital.”	M1		1
“- eu vou fazer a cirurgia, talvez, eu penso muitos nos riscos, sabe...eu tenho medo de ficar imperfeito...não ficar do jeito que eu quero (referindo-se à genitália).”	H2		1

“- Ah....quero prótese mamária e da redesignação sexual.”	M2		1
“- Eu quero fazer redesignação sexual.”	H7		1
“-...eu pretendo fazer a mudança de sexo...pretendo botar prótese nos seios.”	M3		1
“- Eu queria fazer a cirurgia redesignação.”	M4		1
“- O que você deseja fazer? -Trocar o sexo.”<br>“- Trocar a parte genital?- Sim, genital.”	M5		2
“- Sim, a redesignação sexual para retirar o pênis né... E colocar a vagina assim.”	M6		1
“-...se eu pudesse fazer, eu faria a de redesignação sexual.”	NB2		1
		TOTAL DE URS	1[illegible]
		Demanda por mastectomia	
“-Quero, mastectomia e tirar o útero.”<br>“ - Fazer a mastectomia. É o que eu mais quero.”	H3		2
“-...eu pretendo fazer...a cirurgia da parte de cima, a mastectomia.”	H4		1
“-...saber que meus seios diminuíram, e daqui a alguns anos, quem sabe até, fazer cirurgia para retirar....”<br>“P: - O que que você quer fazer de cirurgia? - Retirada dos seios	H5		2

"- O que você quer fazer?- Mastectomia. Só."	H6		1
"- Eu pretendo fazer a mastectomia masculinizadora... "<br>"- eu acho que a mastectomia é um ponto meio que crucial assim"	H8		2
"-...a retirada da mama e útero para evitar um câncer eu quero fazer..."	H9		1
"– Deseja fazer qual?– Tirar os seios."	H10		1
"- Em relação a parte de ser trans, só a mastectomia."	H11		1
"- Você deseja alguma cirurgia? - A retirada dos seios."	H12		1
"- Quero fazer a mastectomia."	H13		1
"-...pretendo fazer a mastectomia..."<br>"-...a mastectomia, somente."	H14		2
"- A mastectomia, só essa."	H15		1
"- Eu tenho vontade de tirar os seios. "	H18		1
"- Tem a questão da retirada da mama. "<br>"- Então a mastectomia pra você é uma questão importante! - Muito, muito!"	H20		2
"- Mastectomia."	H22		1

“-Só mastectomia mesmo.- Útero e ovário não? –Não é uma vontade minha.”	H23		1
“- Você tem demanda cirúrgica? Vontade fazer alguma cirurgia…-<br>Ah, só mastectomia mesmo. “	H24		1
“-...mastectomia.”	H25		1
“- Pretendo fazer mastectomia, só mastectomia.”	H26		1
“- E a única seria a questão do seios?<br>– Sim. Sim, seria sim.”	H28		1
“-...dá pra ver entendeu, que tem seios aqui, então isso me incomoda muito...”	NB2		1
		TOTAL DE URS	2[illegible]
		Demanda por histerectomia	
“- Você quer fazer histerectomia? - Quero, quero...”	H2		1
“- Quero, mastectomia e tirar o útero.”	H3		1
“- ... Depois, futuramente, eu quero fazer a histerectomia.”	H8		1
“-...a retirada da mama e útero para evitar um câncer eu quero fazer...”	H9		1
		TOTAL DE URS	4

		Desinteresse por cirurgia	
“-...não tenho muito interesse em fazer.”	NB1		1
“- Você deseja alguma cirurgia? - Não.”	H17		1
“- No momento, não.”	M7		1
“- Não quero operar não. “	H19		1
“ - Voce tem alguma demanda pra cirurgia, tem vontade de fazer? Mama, nada disso?<br>- Não.”	M8		1
“-...eu não quero tirar meu peito.”	H21		1
“- Pretende fazer algum dia? - Não!”	M13		1
		TOTAL DE URs	7

Total de URs: 216
