# Supplementary material for: Transgender people’s knowledge about the adverse effects of cross-hormonization: challenges for nursing
Source: Rev Bras Enferm. 2024 Sep 20;77(4):e20230346. doi: 10.1590/0034-7167-2023-0346 (PMC11419685; doi:10.1590/0034-7167-2023-0346)
Supplement: 0034-7167-reben-77-04-e20230346-suppl03 [file 0034-7167-reben-77-04-e20230346-suppl03.pdf]

PARECER CONSUBSTANCIADO DO CEP

DADOS DA EMENDA

Título da Pesquisa: Gênero, sexualidade, diversidade e direitos sexuais e reprodutivos: acesso, inclusão, promoção e educação em saúde na região metropolitana do Rio de Janeiro.

Pesquisador: Cláudia Regina Santos Ribeiro

Área Temática:

Versão: 4

CAAE: 10003219.6.0000.5243

Instituição Proponente: Instituto de Saúde Coletiva da UFF

Patrocinador Principal: Financiamento Próprio

DADOS DO PARECER

Número do Parecer: 4.043.089

Apresentação do Projeto:

Trata-se de emenda referente ao projeto "Gênero, sexualidade, diversidade e direitos sexuais e reprodutivos: acesso, inclusão, promoção e educação em saúde na região metropolitana do Rio de Janeiro", referente à solicitação de alteração de cronograma.

Esse projeto tem como temas centrais e objetos de investigação as ações de saúde e de formação profissional voltadas à promoção do acesso, cuidado e dos direitos sexuais e reprodutivos das populações LGBTI - lésbicas, gays, bissexuais, travestis, mulheres e homens transexuais e pessoas intersexo e de homens heterossexuais cisgênero, numa perspectiva de gênero na região metropolitana do Rio de Janeiro. Os participantes da pesquisa serão os/as profissionais de saúde de diversos níveis e tipos de atuação e usuários/as pertencentes a essas populações. O projeto justifica-se pela escassez de pesquisas, ações de saúde e investimentos em formação profissional sobre a saúde dessas populações, transformando as poucas experiências em universos pródigos para essa investigação. Seus resultados poderão colaborar para a ampliação das discussões, melhoria desses serviços de saúde e orientação de outros novos. Como opção metodológica, será

usada a abordagem qualitativa de cunho etnográfico. E para a análise dos dados, serão usados os preceitos norteadores do método hermenêutico-dialético.

O projeto inicial foi aprovado em 08 de abril de 2019 (parecer deste CEP n° 3.250.583) e uma emenda autorizando a inclusão de membros a equipe e aumento do tamanho amostral, em 05 de abril de 2020 (parecer deste CEP n° 3.955.052).

Objetivo da Pesquisa:

Objetivo Primário: Identificar, conhecer e analisar ações de saúde e de educação permanente que visem a melhoria do acesso e do cuidado voltados para os homens heterossexuais cisgêneros e a população LGBTI na região metropolitana do RJ.

Objetivos específicos:

a) Identificar e analisar as ações que visem a promoção da paternidade participativa e cuidadora e seus impactos na saúde dos homens, considerando as diversas possibilidades de masculinidades e paternidades;

b) Conhecer as demandas de saúde da população trans, assim como os facilitadores e as barreiras para a sua satisfação nos serviços de saúde;

c) Analisar os dois primeiros anos de funcionamento do Ambulatório de Saúde Trans João W. Nery;

d) Analisar os impactos das ações de educação permanente na qualidade da atenção a essas populações e na formação dos profissionais de saúde;

e) Produção e publicação de artigos e apresentação dos resultados de pesquisa para os/as participantes.

Avaliação dos Riscos e Benefícios:

Riscos: O risco de vazamento de informações do projeto será neutralizado pelas seguintes ações: uso de gravador e computador pessoais da pesquisadora protegidos por senha. Sobre o risco de desconforto físico e emocional dos/as participantes, os locais das entrevistas serão escolhidos em comum acordo entre as partes visando a privacidade e a garantia do anonimato, assim como o horário e o dia da entrevista. Também será garantido ao/à participante a interrupção da entrevista quando bem quiser, assim como o desligamento da pesquisa respeitando as normas de pesquisa.

Benefícios: A pesquisa poderá beneficiar os/as participantes colaborando para a melhoria dos serviços de saúde onde são atendidos e/ou exercem suas funções profissionais. Poderá também ser um canal de fala para usuários e profissionais tornarem suas demandas conhecidas, colaborando para a promoção da saúde dessas populações e melhoria dos serviços, inclusive para os profissionais.

Comentários e Considerações sobre a Pesquisa:
O presente parecer refere-se à emenda do projeto “Gênero, sexualidade, diversidade e direitos sexuais e reprodutivos: acesso, inclusão, promoção e educação em saúde na região metropolitana do Rio de Janeiro”, com o objetivo de solicitar prorrogação do prazo de execução do projeto até 31 de março de 2021.

A pesquisa é bastante atual e de fácil execução e analisa as ações de saúde e educação permanente que visam a melhoria do acesso e do cuidado voltados para os homens heterossexuais cisgêneros e a população LGBTI.

Na emenda aprovada anteriormente, referente à inclusão de pesquisadores e aumento do tamanho amostral, o colegiado do presente CEP observou que, no projeto aprovado, o cronograma indicava finalização do projeto em dezembro de 2019, com a etapa de entrevistas finalizando em agosto de 2019. Dessa forma, uma vez que o projeto ainda se encontra em andamento, foi orientado que os pesquisadores apresentassem novo cronograma atualizado, solicitando prorrogação do prazo de execução do projeto em nova emenda, apresentando as justificativas cabíveis.

Na presente emenda, na seção “Justificativa da Emenda”, os pesquisadores informaram: “Solicito a prorrogação do prazo de execução do projeto para 31 de Março de 2021, conforme cronograma atualizado.”

Dessa forma, não foram apresentados os motivos que levaram a necessidade de alteração do cronograma, que deveriam ter sido elencados pelos próprios pesquisadores. Entretanto, considerando as informações que os pesquisadores forneceram no relatório parcial, aprovado em

05 de abril de 2020 (parecer deste CEP n° 3.955.045), pudemos avaliar o andamento do projeto e proceder a avaliação da solicitação da presente emenda.

Considerações sobre os Termos de apresentação obrigatória:

Foi apresentado cronograma atualizado, com proposta de finalização do projeto em 31 de março de 2021.

Recomendações:

Vale a pena ressaltar que, no momento de submissão de uma emenda, todas as justificativas devem estar elencadas no documento em questão.

Conclusões ou Pendências e Lista de Inadequações:

O colegiado deste CEP, de acordo com as atribuições definidas na Resolução CNS nº 466 de 2012 e na Norma Operacional nº 001 de 2013 do CNS, manifesta-se pela aprovação desta emenda.

Este parecer foi elaborado baseado nos documentos abaixo relacionados:

Tipo Documento	Arquivo	Postagem	Autor	Situação
Informações Básicas do Projeto	PB_INFORMAÇÕES_BÁSICAS_1537782_E2.pdf	09/04/2020 12:33:59		Aceito
Outros	Relatorioparcial1.docx	18/02/2020 16:11:31	Cláudia Regina Santos Ribeiro	Aceito
Parecer Anterior	ultimoPBPARECERCONSUBSTANCIADOCEP3561873.pdf	03/12/2019 12:44:59	Cláudia Regina Santos Ribeiro	Aceito
Outros	CartaaoCEP.docx	03/12/2019 12:42:47	Cláudia Regina Santos Ribeiro	Aceito
Projeto Detalhado / Brochura Investigador	ProjetodePesquisa.doc	03/12/2019 12:25:17	Cláudia Regina Santos Ribeiro	Aceito
Outros	Roteirosdeentrevistas.docx	08/03/2019 13:43:10	Cláudia Regina Santos Ribeiro	Aceito
TCLE / Termos de Assentimento / Justificativa de Ausência	TECLE.doc	08/03/2019 13:40:06	Cláudia Regina Santos Ribeiro	Aceito
Cronograma	CronogramaPesquisa.docx	08/03/2019 13:39:08	Cláudia Regina Santos Ribeiro	Aceito
Declaração de Instituição e	Anuencia.pdf	26/02/2019 10:41:50	Cláudia Regina Santos Ribeiro	Aceito

Endereço: Rua Marquês de Paraná, 303 - 4º Andar (Prédio Anexo)
Bairro: Centro **CEP:** 24.033-900
UF: RJ **Município:** NITEROI
Telefone: (21)2629-9189 **Fax:** (21)2629-9189 **E-mail:** etica.ret@id.uff.br

Infraestrutura	Anuencia.pdf	26/02/2019 10:41:50	Cláudia Regina Santos Ribeiro	Aceito
Declaração de Instituição e Infraestrutura	Cartadeapresentacaoprojeto.pdf	06/01/2019 10:54:34	Cláudia Regina Santos Ribeiro	Aceito
Folha de Rosto	FolhaRosto.pdf	06/01/2019 10:51:35	Cláudia Regina Santos Ribeiro	Aceito

Situação do Parecer:
Aprovado

Necessita Apreciação da CONEP:
Não

NITEROI, 22 de Maio de 2020

Assinado por:
Adriana Rocha Brito
(Coordenador(a))
