# Supplementary material for: Transgender people’s knowledge about the adverse effects of cross-hormonization: challenges for nursing
Source: Rev Bras Enferm. 2024 Sep 20;77(4):e20230346. doi: 10.1590/0034-7167-2023-0346 (PMC11419685; doi:10.1590/0034-7167-2023-0346)
Supplement: 0034-7167-reben-77-04-e20230346-suppl04 [file 0034-7167-reben-77-04-e20230346-suppl04.pdf]

APÊNDICE B

QUADRO DE SATURAÇÃO TEÓRICA

Participantes* / Unidades de Significação (US)	H** 1***	H 2	M 1	NB 1	H 3	H 4	H 5	H 6	M 2	H 7	H 8	M 3	M 4	H 9	H 10	H 11	M 5	M 6	H 12	H 13	H 14	H 15	H 16	H 17	H 18	M 7	H 19	M 8	H 20	H 21	M 9	H 22	H 23	H 24	M 10	H 25	H 26	M 11	M 12	M 13	NB 2	H 27	H 2[illegible]
Hormonização sem prescrição médica		**x**	x						x	x		x		x			x												x		x	x		x					x	x			
Nenhuma hormonização anterior						**x**		x			x		x		x	x		x		x	x		x		x	x	x	x		x						x	x				x	x	x
Hormonização com prescrição médica	**x**	x		x		x	x		x												x	x						x		x			x		x			x	x				
Oscilações de humor		**x**			x		x			x																				x													
Estresse											**x**			x																					x								
Risco de desenvolvimento de câncer			**x**			x				x				x			x												x														
Problemas Cardiovasculares							**x**		x			x										x				x					x			x									
Cefaleia									**x**																					x													
Depressão				**x**							x																							x									
Infertilidade																																	**x**										
Enjoo									**x**																					x													
Alterações cutâneas						**x**				x	x												x	x											x								
Aumento do apetite								**x**						x																x													
Aumento de peso				**x**																			x																				
Calor								**x**		x																									x			x					
Alopecia						**x**																	x		x								x										
Alteração na quantidade e distribuição dos pelos				**x**		x		x																																			
Alterações na libido			**x**	x					x	x		x																		x	x		x		x			x					
Tabagismo****					**x**						x			x		x				x	x			x			x		x	x			x				x	x			x		x
Aspirações reprodutivas de forma natural																**x**								x					x	x			x	x		x							
Aspirações reprodutivas de forma assistida		**x**			x		x							x	x							x											x										x
Adoção		**x**			x	x				x					x	x			x	x	x				x			x					x				x						
Desinteresse por reprodução			**x**					x			x																x											x		x			
Demanda por prótese mamária			**x**						x			x																			x				x			x					
Demanda por redesignação sexual		**x**	x						x	x		x	x				x	x																							x		
Demanda por mastectomia					**x**	x	x	x			x			x	x	x			x	x	x	x			x				x			x	x	x		x	x				x		x
Demanda por histerectomia		**x**			x						x			x																													
Desinteresse por cirurgia				**x**																				x		x	x	x		x										x			

* As entrevistas encontram-se na ordem em que foram realizadas.
** A letra H significa Homem; a letra M corresponde a Mulher e NB quer dizer Não Binário.

*** O número ao lado da letra corresponde à ordem em que cada homem, mulher ou não binário foi entrevistado(a).
**** O tabagismo não é efeito adverso, mas um potencial fator de risco para os efeitos adversos da hormonização cruzada
