# Supplementary material for: Transgender people’s knowledge about the adverse effects of cross-hormonization: challenges for nursing
Source: Rev Bras Enferm. 2024 Sep 20;77(4):e20230346. doi: 10.1590/0034-7167-2023-0346 (PMC11419685; doi:10.1590/0034-7167-2023-0346)
Supplement: 0034-7167-reben-77-04-e20230346-suppl05 [file 0034-7167-reben-77-04-e20230346-suppl05.pdf]

APÊNDICE C

Quadro síntese das Unidades de Registro e Unidades de Significação na Análise de Conteúdo

		Número de Unidades de Registro (UR)																																											Total UR	T co
**Código**	**Unidades de Significação (US)**	C*1	C2	C3	C4	C5	C6	C7	C8	C9	C10	C11	C12	C13	C14	C15	C16	C17	C18	C19	C20	C21	C22	C23	C24	C25	C26	C27	C28	C29	C30	C31	C32	C33	C34	C35	C36	C37	C38	C39	C40	C41	C42	C43		
1	Hormonização sem prescrição médica		2	3						1	1		1		1			1												2		1	2		2					1	1				20	
2	Nenhuma hormonização anterior						1		1			1		1		2	1		1		1	1		1		1	2	1	1		1						1	1				1	1	1	22	[illegible]
3	Hormonização com prescrição médica	1	1		1		1	1		1												1	1						1		1			1		1			2	1					15	
4	Oscilações de humor		2			1		1			1																				1														6	
5	Estresse											2			1																					1									4	
6	Risco de desenvolvimento de câncer			1			1				1				1			1												2															7	
7	Problemas Cardiovasculares							3		1			1										1				1					1			1										9	
8	Cefaleia									2																					1														3	
9	Depressão				1							1																							1										3	
10	Infertilidade																																	2											2	
11	Enjoo									1																					1														2	
12	Alterações cutâneas						1				1	1												1	1											2									7	
13	Aumento do apetite								1						1																1														3	
14	Aumento de peso				1																			1																					2	
15	Calor								1		1																									1			1						4	
16	Alopecia						1																	1		1								1											4	
17	Alteração na quantidade e distribuição dos pelos				1		1		1																																				3	
18	Alterações na libido			1	1					1	1		1																		1	1		1		1				1					10	
19	Tabagismo					1						1			1		1				1	1			1			1		1	1			1				1	1			1		1	15	
20	Aspirações reprodutivas de forma natural																1								2					1	2			1	1		1								9	
21	Aspirações reprodutivas de forma assistida		2			1		1							1	1							1											1										1	9	
22	Adoção		1			1	1				1					1	1			1	1	1				1			1					1				1							13	
23	Desinteresse por reprodução			1					1			1																1											1		1				6	
24	Demanda por prótese mamária			1						1			1																			1				1			1						6	
25	Demanda por redesignação sexual		1	1						1	1		1	1				2	1																							1			10	
26	Demanda por mastectomia					2	1	2	1			2			1	1	1			1	1	2	1			1				2			1	1	1		1	1				1		1	26	[illegible]
27	Demanda por histerectomia		1			1						1			1																														4	

28	Desinteresse por cirurgia				1																				1		1	1	1		1										1				7	

* C = Corpus

TOTAL UR: 231
