# Supplementary material for: Transgender people’s knowledge about the adverse effects of cross-hormonization: challenges for nursing
Source: Rev Bras Enferm. 2024 Sep 20;77(4):e20230346. doi: 10.1590/0034-7167-2023-0346 (PMC11419685; doi:10.1590/0034-7167-2023-0346)
Supplement: 0034-7167-reben-77-04-e20230346-suppl06 [file 0034-7167-reben-77-04-e20230346-suppl06.pdf]

Guia de pesquisa sobre saúde da população transgênero

Informações dos autores
Autor Principal
Nome: Andréa Felizardo Ahmad
ORCID: 0000-0002-1269-3612
Instituição: Universidade Federal do Estado do Rio de Janeiro (UNIRIO)
E-mail: andreafelizardo.enfermeira@gmail.com

Contato alternativo / Co-autor
Adriana Lemos / UNIRIO
ORCID: 0000-0001-9705-6200
Instituição: Universidade Federal do Estado do Rio de Janeiro (UNIRIO)
E-mail: adrianalemmos@unirio.br

Data da coleta de dados: 2019 – 07- 01 a 2020 – 02 – 28.
Localização da coleta de dados – Niterói, Rio de Janeiro, Brasil.

O conjunto de dados possui os seguintes arquivos:

- Ap. B – Quadro de Saturação Teórica

Este arquivo 2020 – 04 - 12, e consiste na compilação de dados, através das falas das pessoas participantes da pesquisa (Unidades de Registro), cujos assuntos em comum foram agrupados em Unidades de Significação, de acordo com a análise de conteúdo de Bardin. Esses dados permitem verificar se os objetivos da pesquisa estão sendo alcançados, ou se ainda mais entrevistas precisam ser feitas para alcançá-los.

- Ap. C – Quadro Síntese UR/US

Este arquivo foi criado em 2020 – 04 – 29, e consiste na apresentação da quantidade de vezes que cada participante apresentou uma Unidade de Registro (fala) sobre determinado assunto (Unidade de Significação).

- Ap E – Caracterização dos participantes

Este arquivo foi criado em 2020 – 03 - 23, e consiste nas informações que caracterizam as pessoas participantes, de acordo com a idade, identidade de gênero, orientação sexual, raça/etnia, escolaridade, profissão, estado conjugal, renda (em salários mínimos) e religião. As pessoas participantes estão identificadas com as letras H (homem) e M (mulher), seguidas de um número de acordo com a ordem em que as entrevistas foram realizadas.

- Fala URs Trans

Este arquivo foi criado em 2020 – 04 - 17, e consiste na transcrição literal das Unidades de Registro (falas) das pessoas participantes, relacionadas aos participantes; agrupadas nas Unidades de Significação e o total de unidades de registro por participante. Além disso, consta, ao final do arquivo, o total de Unidades de Registro encontradas. Este arquivo integra o quadro síntese UR / US.

- Parecer CEP

Este parecer foi emitido em 2019 – 04 - 08, e consiste no Parecer emitido pelo Comitê de Ética em Pesquisa da Universidade Federal Fluminense, a fim de que a pesquisa pudesse ser desenvolvida no campo de estudo escolhido.
