# Supplementary material for: Transgender people’s knowledge about the adverse effects of cross-hormonization: challenges for nursing
Source: Rev Bras Enferm. 2024 Sep 20;77(4):e20230346. doi: 10.1590/0034-7167-2023-0346 (PMC11419685; doi:10.1590/0034-7167-2023-0346)
Supplement: Translation

# Translation: Conhecimento de pessoas transgênero sobre os efeitos adversos da hormonização cruzada: desafios para a enfermagem

**DOI:** 10.1590/0034-7167-2023-0346pt

**Authors:** Andréa Felizardo Ahmad, Adriana Lemos, Cláudia Regina Ribeiro, Luciane Marques de Araujo, Vanessa de Almeida Ferreira Corrêa, Janaína Pinto Janini, Rosâne Mello, Beatriz Rodrigues Silva Selles Dantas

**Affiliations:** I Universidade Federal do Estado do Rio de Janeiro. Rio de Janeiro, Rio de Janeiro, Brasil; II Universidade Federal Fluminense. Niterói, Rio de Janeiro, Brasil; III Universidade do Estado do Rio de Janeiro. Rio de Janeiro, Rio de Janeiro, Brasil; IV Centro Universitário IBMR. Rio de Janeiro, Rio de Janeiro, Brasil; V Secretaria Municipal de Maricá. Maricá, Rio de Janeiro, Brasil

**Keywords:** Conhecimento, Efeitos Adversos, Enfermagem, Hormônios Esteroides Gonadais, Transexualidade

## Abstract

**Objetivos:**

identificar o conhecimento de mulheres e homens trans sobre os efeitos adversos da hormonização cruzada e compreender as repercussões das práticas da hormonização na saúde de mulheres e homens trans.

**Métodos:**

pesquisa exploratória, descritiva, qualitativa, desenvolvida com 41 participantes, no período de julho de 2019 a fevereiro de 2020, em um ambulatório de saúde trans. Utilizou-se a análise de conteúdo temático-categorial.

**Resultados:**

a partir da análise, emergiram as categorias: Conhecimento sobre os efeitos adversos da hormonização cruzada; e Práticas da hormonização cruzada e seu significado.

**Considerações Finais:**

as práticas de enfermagem, a partir da identificação do conhecimento sobre os efeitos adversos e da compreensão das práticas da hormonização cruzada na saúde das mulheres e dos homens trans, podem se concretizar no cuidado mais inclusivo.

## INTRODUÇÃO

A percepção da necessidade de investigar o conhecimento de mulheres e homens trans sobre os efeitos adversos da hormonização cruzada surgiu a partir da observação, de forma empírica, de que parte da população transgênero, em razão da dificuldade de acesso à saúde e em busca de afirmar suas escolhas, recorria a recursos terapêuticos perigosos, sendo um deles a automedicação. Perante esses fatos, fez-se necessário pesquisar se as pessoas transgênero, que fazem uso de hormônios, conhecem as repercussões que essas substâncias podem trazer à sua saúde.

Nesse contexto, a transição entre os gêneros é o que caracteriza a pessoa transgênero como a pessoa que possui a identidade de gênero diferente do gênero atribuído, de acordo com a sua genitália, ao nascimento. A concordância entre a identidade de gênero e o sexo definido ao nascimento designa a pessoa cisgênero, também chamada de mulher ou homem cis^(1)^.

Ressalta-se que a travesti também é uma pessoa trans, pois ela transita entre os gêneros; nasceu biologicamente homem e tem, na mesma área corporal, nuances femininas e masculinas, todavia a travesti refere-se a si no gênero feminino e constrói seu corpo de acordo com esse gênero^(2)^.

Durante a década de 1980, a epidemia de HIV/AIDS (Vírus da Imunodeficiência Humana/Síndrome da Imunodeficiência Adquirida) fortaleceu os movimentos sociais em busca de políticas públicas para a população LGBT, e a consolidação dessas políticas ocorreu em razão da união entre os membros. Porém, houve relutância em reunir, em um mesmo grupo, o movimento homossexual e as travestis, integrando na sigla a letra T, pois, até então, o movimento era intitulado Movimento Homossexual Brasileiro, abrangendo apenas *gays* e lésbicas. Em 1995, houve a participação oficial das travestis, sendo acrescida a letra T. A letra, então, se referia apenas a elas, pois a inclusão das pessoas transgênero só ocorreu nos anos 2000^(3)^.

A representação social das travestis passa pela violação de acordos políticos dentro do movimento social com a inclusão das mulheres transgênero, tendo em vista a discrepância social entre ambas. Essa violação de acordos políticos está vinculada, segundo as travestis, ao fato de as mulheres transgênero estarem menos comprometidas com a militância política e mais preocupadas com a mudança de seus corpos através do processo transexualizador^(4)^.

Considerando a transição entre os gêneros, o hormônio indicado para mulheres transgênero é o estrogênio, a fim de desenvolver os caracteres secundários femininos, sendo os mais desejados o afinamento do rosto, a obtenção de formas corporais arredondadas e o aumento das mamas, entre outras mudanças corporais. Nos homens trans, é empregada a testosterona, cujo propósito é formar os caracteres secundários masculinos, sendo os mais almejados o agravamento da voz, o surgimento de pelos, entre outros. A hormonização deve ter cuidadoso acompanhamento profissional, diante dos fatores de risco associados. Dessa forma, a hormonização cruzada não é recomendada e adotada apenas como etapa anterior às cirurgias de redesignação sexual, mas por vezes é um processo que encerra a busca pelas mudanças desejadas^(5-6)^.

O presente trabalho justifica-se diante da possibilidade de qualificar as ações de enfermagem, a partir da identificação do conhecimento das pessoas transgênero sobre os efeitos adversos relacionados à hormonização cruzada. Percebe-se, assim, a relevância deste estudo para a população trans por meio da contribuição da enfermagem nas pesquisas científicas e da atenção em saúde qualificada e segura para as pessoas transgênero.

## OBJETIVOS

Identificar conhecimento de mulheres e homens trans sobre os efeitos adversos da hormonização cruzada e compreender as repercussões das práticas da hormonização na saúde de mulheres e homens trans.

## MÉTODOS

### Aspectos éticos

O presente artigo é um recorte da Dissertação de Mestrado, intitulada “Ah! Sei lá, só quero ser eu: significados, saberes e práticas da hormonização cruzada na saúde de mulheres e homens trans”, apresentada ao Programa de Pós-Graduação da Escola de Enfermagem Alfredo Pinto, da Universidade Federal do Estado do Rio de Janeiro (UNIRIO), Rio de Janeiro, Brasil^(7)^.

Em cumprimento às questões éticas em pesquisa com seres humanos, este estudo foi aprovado por Comitê de Ética em Pesquisa, cumprindo todos os critérios e cuidados para a realização de pesquisas envolvendo seres humanos no Brasil, regulamentados pelas Resoluções n^o^ 466/2012 e n^o^ 510/2016 do Conselho Nacional de Saúde.

As(os) participantes, após serem informadas(os) sobre a justificativa, os objetivos e a metodologia do estudo, assinaram um Termo de Consentimento Livre e Esclarecido, em linguagem clara e objetiva, com as informações essenciais para facilitar a compreensão de todas e todos. A confidencialidade dos dados e o respeito ao anonimato foram assegurados através de codinomes com as letras “M” (mulher transgênero) e “H” (homem transgênero), seguidas de números (M1, H2 etc.), de acordo com a ordem de realização das entrevistas.

### Tipo de estudo

Trata-se de pesquisa exploratória, descritiva, com abordagem qualitativa. O estudo foi conduzido em conformidade com a diretriz de relatórios *COnsolidated criteria for REporting Qualitative research* (COREQ)^(8)^.

### Cenário do estudo

O estudo foi realizado em um ambulatório de saúde trans, componente de uma policlínica do serviço público do município de Niterói, RJ, Brasil. O fator geográfico foi o motivo de escolha desse cenário. No referido ambulatório, prestam assistência à população transgênero uma assistente social, um endocrinologista e um psicólogo. Há de se enfatizar que as autoras deste estudo não possuem qualquer vínculo com a instituição do cenário do estudo, porém a pesquisadora principal possui experiência no acolhimento de pessoas trans.

### Fonte de dados

Participaram desta pesquisa 13 mulheres transgênero e 28 homens trans, e nenhuma das pessoas participantes identificou-se como travesti. Os critérios de elegibilidade para as(os) participantes da presente pesquisa foram mulheres e homens trans com idade acima de 18 anos e que fossem usuárias(os) do referido ambulatório, cuja seleção ocorreu de forma aleatória simples. Foram excluídas, deste estudo, as pessoas que se identificaram como não-binárias, por não contemplarem os objetivos desta investigação.

### Coleta e organização dos dados

Primeiramente, foi realizada uma aproximação com o cenário, por meio de uma visita técnica, na qual ocorreram diálogos com as(os) profissionais atuantes no cenário do estudo e com usuárias e usuários do serviço. O trabalho de campo iniciou-se em julho de 2019, e o término foi em fevereiro de 2020.

Para a obtenção dos dados, as(os) participantes foram conduzidas(os) para uma sala privativa, onde ocorreu a entrevista semiestruturada, utilizando um instrumento de coleta de dados com 11 questões pertinentes aos objetivos deste estudo, além de perguntas referentes à caracterização das pessoas participantes. As entrevistas foram audiogravadas e posteriormente transcritas.

### Análise dos dados

Para o tratamento dos dados, utilizou-se a análise de conteúdo temático-categorial, iniciada pela leitura flutuante de todas as entrevistas e, em seguida, foram construídas as hipóteses provisórias^(9-10)^. Após a transcrição de cada entrevista, essas foram finalizadas na de número 41, momento em que constatou-se a ausência de novos fenômenos e que percebeu-se a robustez dos dados, a fim de atingir os objetivos propostos.

Após a leitura flutuante das entrevistas, as Unidades de Registro (UR) foram elaboradas de acordo com as falas das(os) entrevistadas(os), totalizando 141 UR, e, após a análise temática, foram agrupadas em 19 Unidades de Significação (US), de acordo com os temas encontrados.

## RESULTADOS

A idade mediana das pessoas participantes foi de 24 anos. No Quadro 1, é possível observar a caracterização socioeconômico e demográfica dos participantes.

**Quadro 1:** Caracterização socioeconômico e demográfica dos participantes da pesquisa

Características	N = 41	Porcentual
**Gênero**		
Mulher transgênero	13	32%
Homem transgênero	28	68%
**Orientação sexual**		
Heterossexual	21	51%
Pansexual	08	20%
Sem orientação sexual	04	10%
Bissexual	03	07%
Homossexual	01	02%
Não quiseram responder	04	10%
**Escolaridade**		
Ensino médio completo	17	41%
Ensino superior incompleto	13	32%
Ensino médio incompleto	04	10%
Ensino fundamental completo	02	05%
Ensino fundamental incompleto	02	05%
Ensino superior completo	01	02%
Não quiseram responder	02	05%
**Raça/etnia autodeclarada**		
Negra	27	66%
Branca	11	27%
Não quiseram responder	03	07%
**Religião**		
Não tem religião	23	56%
Católica	06	15%
Umbandista	05	12%
Candomblecista	04	10%
Evangélica	01	02%
Não quiseram responder	02	05%
**Estado conjugal**		
Solteira/o	27	66%
União estável	07	17%
Casada/o	06	15%
Separada/o	01	02%
**Renda familiar**		
< que um salário mínimo	01	02%
01 a 02 salários mínimos	07	17%
02 a 04 salários mínimos	07	17%
> 04 salários mínimos	02	05%
Renda variável	05	12%
Não quiseram responder	19	46%

Observou-se que, quanto à orientação sexual, predominou a heterossexualidade. No que se refere à escolaridade, a maioria dos participantes informou que completou o ensino médio. Quanto à raça/etnia, prevaleceu a autodeclaração da raça negra. Sobre a religião, o maior número de participantes afirmou não ter religião. A respeito do estado conjugal, a maioria denominou-se solteiras(os), e quanto à renda familiar, houve predomínio da renda de até quatro salários mínimos.

Houve diversidade de ocupações/profissões entre as(os) participantes, porém há de se destacar que 29,82% relataram não ter nenhuma ocupação no momento da entrevista.

Há de se destacar que o consumo de tabaco foi mencionado por 50% dos homens trans, e 15,38% das mulheres transgênero relataram o uso da substância.

A partir da articulação das falas das pessoas participantes, originaram-se categorias a seguir:

### Categoria 1 - Conhecimento sobre os fatores de risco da hormonização cruzada

Essa categoria foi construída a partir da articulação de 84 UR do *corpus* de análise, originando 16 US, com destaque para o risco de problemas cardiovasculares e o risco de desenvolvimento de câncer. Sobre os efeitos adversos da hormonização cruzada, 78% dos participantes declararam conhecer alguns efeitos, porém algumas pessoas não demonstravam preocupação com esses possíveis danos:

*A gente, no meio trans, tem muito isso, de se autoindicar uma pra outra, sabe. A última coisa que vem na cabeça é que pode fazer mal.* (M4)

*Comecei com 16 anos tomando, sei lá, uma ampola por semana, duas, sei lá* […] *eu só queria um resultado rápido, e o que eu sempre li desde o início quando eu comecei a estudar é que a gente tem que retirar o útero ou ovário, porque isso pode dar câncer no colo do útero, é o que dizem.* (H20)

*Como é que eu vou num médico? Ele não vai me reconhecer como um homem trans* […] *eu já tive uma cólica tão grande, que chorava de dor* […]*, e o médico fala, um cara grandão desse, chorando de dor* […] *tanta dor, que minha pressão até aumentava, e eu sou hipertenso!* (H24)

A respeito do risco de desenvolvimento de câncer, a sua correlação com hormonização cruzada foi apontada como uma preocupação, como se vê nas falas abaixo:

*Eu fiquei com medo de iniciar a transição e ter lá pra frente algum câncer de mama, de útero e tal.* (H09)

*Tem umas amigas minhas que teve esse negócio de câncer* […] *aí eu fiquei com medo, fiquei sabendo que pode dar câncer* […] *nos negócios* [pênis] *da gente.* (M01)

Salienta-se que, ainda que o corpo trans tenha algumas singularidades, a identidade de gênero não pode ser considerada um impeditivo para o cuidado em saúde, de forma que o acolhimento e o atendimento a todas as pessoas precisam ocorrer de forma igualitária e equânime. Apesar disso, há homens trans que mencionam motivos diversos para não procurarem assistência ginecológica, como observado a seguir:

*Sempre tive pavor de ir na ginecologista. Cheguei a ir, mas, naquele primeiro contato, que eu sentia um pouco de falta de empatia, falta de conhecimento* […] *em alguns casos, até um pouco de discriminação* […] *então eu simplesmente levantava da cadeira e ia embora, eu não queria ser atendido.* (H09)

*Eu não quero, porque eu mal consigo me olhar no espelho, por que, né? Eu sou diferente. Outras pessoas verem o que eu não quero ver, pra mim, seria ainda mais um problema. E hoje em dia, eu não sei se algum dia na minha vida eu vou conseguir ir num ginecologista.* (H04)

Diante dos fatos relatados, compreende-se que, através do vínculo das pessoas transgênero com o serviço de saúde, é possível identificar precocemente os efeitos adversos da hormonização cruzada que podem resultar em danos à saúde.

### Categoria 2 - Práticas da hormonização cruzada e seu significado: “Eu quero poder me olhar e me ver da forma que eu me imagino!”

Essa categoria representou um total de 57 UR do *corpus* de análise, agrupadas em três US: nenhuma hormonização anterior; hormonização sem prescrição médica; e hormonização com prescrição médica. Todas as pessoas entrevistadas declararam o desejo da hormonização cruzada e do total de participantes, e 19 relataram nunca terem usado hormônio por meios próprios, sendo 04 mulheres transgênero e 15 homens trans. Das(os) que faziam uso, 13 relataram sua utilização sem prescrição médica e 14 iniciaram ou planejavam iniciar a hormonização com prescrição.

Alguns homens trans estavam emocionados, após receberem a prescrição médica para iniciar a hormonização cruzada, como se percebe nas falas a seguir:

*Ah, eu não tenho palavras! Acho que nunca pensei que pedaços de papéis fossem me fazer tão feliz.* (H01)

*Poxa, foi muita felicidade, porque eu já tava esperando por isso há muito tempo, né, naquela ansiedade de começar minha terapia, a transformação, né?* (H05)

Já para as mulheres transgênero, o acesso à hormonização teve significado diferente, pois elas ficavam felizes ao receber a prescrição, porém sem a euforia e alegria explícitas dos homens, visto nas falas abaixo:

*Agora que vou começar a tomar o medicamento que o médico receitou.* (M02)

*Eu vim procurar o endócrino pra fazer a terapia hormonal e agora tô procurando um psicólogo.* (M08)

Esse fato pode estar relacionado ao acesso mais facilitado aos hormônios, pois a aquisição de hormônios femininos passa antes, pela busca de informações, sobretudo através de outras mulheres transgênero:

*Ali ninguém receita nem passa remédio pra ninguém. É troca de conhecimento, ah, eu tomei o remédio tal. Outros fazem tratamento com endócrino: “Ah, meu endócrino passou isso”. É troca de experiência, na verdade. Só que, aí eu, louca do jeito que eu sou, eu fui e pensei: “Fulana tomou isso e ficou legal, vou tomar também isso daí”.* (M09)

No caso dos homens, mesmo com as dificuldades ocasionadas pelo fato de que a testosterona só pode ser adquirida com prescrição médica, há homens transgênero que conseguem adquirir esse hormônio pela internet ou em academias, como vemos nos discursos abaixo:

*Entrei* [na internet], *e comecei a ver que os homens trans que tinham o mesmo biotipo que o meu e em quanto tempo tomavam o medicamento, e comecei a fazer o mesmo ciclo.* (H09)

*Eu fazia tratamento hormonal em casa mesmo. Eu comprava em academia, porque não tinha ambulatório, eu não tinha acesso a isso.* (H22)

Sobre o uso de hormônios sem prescrição médica, seis mulheres transgênero e 15 homens trans mencionaram nunca ter usado hormônio por meios próprios, mesmo tendo facilidade de acesso:

*Eu nunca gostei dessas coisas* [automedicação], *porque eu sempre fui cabeça nesse sentido. Eu nunca gostei de me medicar sem recomendação médica.* (M06)

Ressaltam-se os riscos da utilização de hormônios sem acompanhamento médico, pois os danos à saúde podem sobrepor os benefícios esperados pela população transgênero.

## DISCUSSÃO

A Resolução nº 47 da Diretoria Colegiada (RDC) da Agência Nacional de Vigilância Sanitária (ANVISA), de 08 de setembro de 2009, define efeito adverso como “qualquer ocorrência médica desfavorável, que pode ocorrer durante o tratamento com um medicamento, mas que não possui, necessariamente, relação causal com esse tratamento”^(11)^.

Nesse contexto, o Ministério da Saúde (MS) é enfático quanto ao esclarecimento da população trans a respeito da hormonização, seu uso prolongado e possíveis efeitos adversos. Dessa forma, o MS objetiva a redução de riscos e danos por uso indiscriminado de medicamentos (hormônios, inclusive) entre a população trans. Há de se oferecer informação contínua e acompanhamento periódico, a fim de minimizar os riscos e resguardar a população transgênero de danos à sua saúde^(12)^.

Sobre o risco de desenvolver problemas cardiovasculares, a trombose foi citada por alguns participantes, mostrando a preocupação com esse perigo iminente. Há de se ressaltar que um dos fatores de risco para doenças cardiovasculares, trombose inclusive, é o consumo de tabaco, o qual foi citado por uma parcela dos participantes da pesquisa. O tabagismo está entre uma das principais causas de doenças cardiovasculares e dos mais variados tipos de câncer. A facilidade de conseguir a droga, devido à sua licitude e ao baixo custo, permite que seu consumo seja desenfreado, ainda que sejam fornecidas as informações sobre as comorbidades^(13)^.

Contudo, o risco de doenças cardiovasculares não está relacionado somente a fatores de risco, tais como o consumo de álcool e tabaco, mas também à hormonização cruzada relacionada a esses fatores, pois essas substâncias, associadas ao uso de testosterona e de valerato de estradiol, podem elevar o risco de tromboembolismo venoso e cardiopatias. O estresse também é um fator de risco para doenças cardiovasculares^(14)^.

Dessa forma, entende-se que o acompanhamento regular por profissionais de saúde, diante dos possíveis efeitos adversos que podem ocorrer durante a hormonização cruzada, pode contribuir para a redução dos fatores de risco cardiovascular^(15)^.

Sobre o risco de desenvolvimento de câncer, estudo australiano evidenciou a ineficácia do rastreio de câncer de colo uterino em homens trans diante de diversos fatores, desde a inabilidade profissional para realizar o exame até o constrangimento do homem trans ao buscar o procedimento, devido à discriminação e à disforia em relação à genitália^(16)^.

Salienta-se que os homens trans não devem ser excluídos da coleta do exame citopatológico (sendo facultativa, de forma geral, a coleta do referido exame em homens trans que nunca tiveram relação sexual com penetração peniana), destacando-se que esse exame tem por finalidade detectar o mais precocemente possível a presença de lesões precursoras de câncer de colo uterino^(2)^.

A respeito da incidência de câncer de mama em homens trans, sua ocorrência é rara, porém eles devem se submeter ao rastreio da doença, conforme preconizado, inclusive homens trans que fizeram mastectomia, assim como é relevante o exame de rastreio para câncer de mama em mulheres transgênero^(17)^.

A discussão sobre a importância do rastreio para a prevenção do câncer é evidenciada diante da incidência de cânceres diversos, tais como neoplasias mamárias, neoplasias relacionadas ao uso de androgênios por homens trans (mesmo àqueles que fizeram mastectomia) e de câncer de próstata, associadas ao uso de estrogênio, por mulheres transgênero. Ainda assim, mais estudos são necessários para que se comprovem as hipóteses formuladas^(18)^.

Entende-se que o preconceito e os pré-julgamentos afastam a(o) enfermeira(o) e outras(os) profissionais de saúde de realizar a assistência de forma acolhedora e resolutiva, comprometendo assim a saúde das pessoas trans no que compete à educação em saúde relacionada aos efeitos adversos da hormonização cruzada. Esse comportamento é uma forma de violência que contribui para que algumas doenças deixem de ser rastreadas devidamente, devido à vulnerabilidade a qual a população trans está exposta^(19)^.

Esses aspectos corroboram o quão é relevante a educação em saúde, diante da inconsistência na obtenção de informações e da discriminação, já implícita na população transgênero. Assim, é imprescindível a capacitação dos profissionais de saúde quanto à saúde da população trans e, dessa forma, propiciar um ambiente acolhedor e respeitoso para o atendimento a esse público^(20)^.

No sentido de promoção da saúde, as barreiras a serem transpostas incluem o desinteresse dos gestores em capacitar as(os) profissionais de saúde e a inércia de algumas enfermeiras e enfermeiros em propor ações de saúde baseadas nas problemáticas que usuárias e usuários poderiam trazer, inclusive dúvidas sobre a hormonização cruzada^(21)^.

Para que haja a percepção dessas demandas, é fundamental o vínculo entre usuária(o) e enfermeira(o) e também a compreensão da Política de Atenção à Saúde LGBT, na qual abordam-se diversas questões, entre elas a hormonização cruzada^(11)^.

Nesse contexto, a educação em saúde, não somente para a população transgênero, mas também para toda a sociedade, pode ser um divisor de águas para a redução da discriminação e do preconceito, fatores esses que dificultam o acesso aos serviços de saúde por mulheres e homens trans.

Estudo realizado em 2017, no Brasil, observou que, das dez mulheres transgênero participantes da pesquisa, oito fizeram uso da hormonização cruzada sem prescrição médica, sendo assim percebida a existência de modificações no comportamento e implicações na fertilidade^(22)^.

A respeito do uso de hormônios sem prescrição médica, os motivos dados pelas(os) participantes do presente estudo para essa prática foram a dificuldade de acesso aos serviços de saúde e o anseio por resultados rápidos. Assim, há de se refletir sobre o fato de mais mulheres transgênero do que homens trans fazerem uso da hormonização cruzada sem prescrição médica, facilitada pela possibilidade de compra do hormônio feminino sem a devida prescrição.

Ademais, obter os hormônios femininos ocorre, muitas vezes, após as considerações obtidas na internet e através de outras mulheres transgênero. Porém, as mudanças corporais ocasionadas pela hormonização cruzada não são suficientes diante das barreiras sociais que as mulheres transgênero vivenciam^(23)^.

As barreiras no acesso aos serviços de saúde e, consequentemente, à prescrição hormonal, estimulam a busca por informações de forma inadequada e o início da hormonização cruzada de forma indiscriminada, colocando a sua saúde em risco^(24)^.

À luz das questões apresentadas, compreende-se que a hormonização mostra-se o veículo para a obtenção rápida, para algumas e alguns, das formas femininas ou masculinas, tão importantes para a individualidade de mulheres e homens trans, o que muitas vezes levam ao uso sem acompanhamento médico, mesmo que se saibam quais são seus efeitos adversos. No entanto, para as(os) participantes desta pesquisa, essa postura não foi a predominante.

### Limitações do estudo

Destaca-se, como limitação do estudo, o fato de a unidade de saúde, que foi cenário do estudo, funcionar somente uma vez por semana, o que implicou questões logísticas quanto ao tempo para que ocorresse a construção dos dados. Contudo, os resultados apresentados corroboram a importância de discutir a temática nos espaços de saúde, trazendo a reflexão da necessidade de mais estudos que abordem questões relacionadas à transexualidade.

Aponta-se, como mais uma limitação do estudo, a ausência de profissionais de enfermagem no cenário do estudo, diante das contribuições assistenciais em saúde, destacando que a enfermagem faz parte da equipe multidisciplinar do processo transexualizador. Ademais, as práticas de assistência de enfermagem, a partir do conhecimento das pessoas transgênero sobre a hormonização cruzada, podem se concretizar no cuidado integral e equânime e em ações educativas em saúde no que diz respeito à importância da hormonização cruzada com acompanhamento de equipe multidisciplinar.

### Contribuições para a área de enfermagem, saúde ou política pública

Por meio deste estudo, foi possível refletir sobre as peculiaridades que mulheres e homens trans enfrentam, a fim de obter cuidados em saúde e ter seus direitos respeitados. Os resultados deste estudo demonstraram que o conhecimento da população trans sobre a hormonização cruzada e as práticas da hormonização cruzada na saúde da população trans são fatores que impactam significativamente na assistência de enfermagem e na atenção à saúde desse público, através da construção de projetos terapêuticos e criação de vínculos, e quanto à prevenção e detecção precoce de efeitos adversos ocasionados pela hormonização, além do rastreio de câncer.

## CONSIDERAÇÕES FINAIS

Este estudo propiciou, através das narrativas dos participantes, a identificação do conhecimento de mulheres e homens trans sobre os efeitos adversos da hormonização cruzada, destacando que a maioria dos participantes declarou conhecer os efeitos adversos da hormonização cruzada através de pesquisas na internet e de informações socializadas em grupos de discussão trans.

Esse conhecimento não impediu que algumas pessoas fizessem uso da hormonização cruzada sem acompanhamento de profissionais de saúde, porém ressalta-se que a busca pelo cuidado em saúde em um ambulatório de saúde integral para a população trans demonstra a preocupação dessas pessoas com a sua saúde, a fim de usufruir do máximo benefício da hormonização, e que os efeitos adversos e riscos inerentes à hormonização pudessem ser rapidamente identificados, ainda que algumas tenham encontrado meios mais fáceis e rápidos de praticar a hormonização cruzada através da automedicação.

Infelizmente, não é possível prever com segurança os efeitos adversos que a hormonização cruzada pode ocasionar em cada pessoa, pois fatores como o tabagismo e o uso dessas substâncias sem acompanhamento profissional podem obscurecer alguns riscos, tais como de desenvolvimento de câncer e de problemas cardiovasculares. Assim, o criterioso acompanhamento pela equipe multidisciplinar se faz necessário, no intuito de prevenir esses possíveis efeitos e proporcionar plena saúde e satisfação de cada pessoa na sua identidade de gênero.

Este estudo permitiu a compreensão das repercussões das práticas da hormonização cruzada na saúde de mulheres e homens trans, pois a busca pelo corpo correspondente ao interesse da mulher ou do homem trans através da hormonização cruzada é uma demanda caracterizada por diversos significados em comum: percorrer os mais diversos espaços (de saúde inclusive) sem enfrentar olhares discriminatórios ou comentários preconceituosos; olhar-se e gostar do que vê, reconhecendo-se e sendo reconhecida ou reconhecido de acordo com a identidade de gênero, em face à aquisição dos caracteres secundários; e ainda obter tranquilidade no seu dia a dia. Outra importante repercussão foi a segurança oferecida pela passabilidade diante da transfobia enfrentada frequentemente pelas pessoas transgênero.

Por fim, destaca-se a contribuição deste estudo para fomentar a pesquisa no âmbito da atenção à saúde da população transgênero, bem como para o ensino de graduação não só para a área de enfermagem, mas também para a saúde em geral. A discussão sobre transexualidade nos espaços de cuidado em saúde, tanto por via de atividades extensionistas, em parceria com universidades, quanto por via de educação permanente, podem contribuir para práticas profissionais mais inclusivas e assim, colaborar para a redução da transfobia que tanto causa danos à saúde dessa população. Dessa forma, este estudo contribui para a minimização dos riscos e danos diante da vulnerabilidade social a que esse público está exposto.

## DISPONIBILIDADE DE DADOS E MATERIAL

*https://doi.org/10.48331/scielodata.HWNTYR*
